# The pest control and pollinator protection dilemma: The case of thiamethoxam prophylactic applications in squash crops

**DOI:** 10.1371/journal.pone.0267984

**Published:** 2022-05-20

**Authors:** Diana Obregon, Grace Pederson, Alan Taylor, Katja Poveda

**Affiliations:** 1 Department of Entomology, Cornell University, Ithaca, New York, United States of America; 2 Biological Sciences, Cornell University, Ithaca, New York, United States of America; 3 Department of Horticulture, Cornell AgriTech, Cornell University, Geneva, New York, United States of America; Institut Sophia Agrobiotech, FRANCE

## Abstract

A major challenge in sustainable agriculture is finding solutions to manage crop-damaging pests such as herbivores while protecting beneficial organisms such as pollinators. Squash is a highly pollinator-dependent crop that is also attractive to herbivores like the striped cucumber beetle. While synthetic insecticides can provide control of insect pests, they can also affect non-target organisms such as pollinators. Thus, growers need to balance pest management with pollinator protection to ensure optimal yield. Thiamethoxam is a commonly used systemic insecticide that translocates throughout plants, leaving residues in nectar and pollen. The aim of this study was to evaluate whether there are uses of this insecticide that provides efficient pest control while minimizing pesticide pollinator exposure. Specifically, we tested how different prophylactic application methods (seed treatments, in-furrow applications, and early foliar sprays) of commercially available thiamethoxam products impact pest control, bee visitation, yield, and pesticide residues in flowers of squash crops. We found that among the different methods of thiamethoxam application, in-furrow application best prevented defoliation and resulted in the highest fruit weight and number. However, it also produced the most frequent and highest concentrations of thiamethoxam in nectar and pollen, reaching lethal levels for squash bees. Our study provides evidence that under current application methods, thiamethoxam does not provide a sustainable solution for squash growers and further research is required on more efficient pesticide delivery methods, as well as non-pesticide pest control measurements.

## 1. Introduction

Global agriculture is becoming increasingly pollinator-dependent due to the expansion of nutrient-dense crops such as oilseeds, fruits, and nuts that greatly benefit from insect pollination to obtain optimal yields [1]. This expansion poses a growing need for pollinators amid the mounting evidence of insect decline [2–4], which threatens the stability of food production systems [5]. Additionally, the benefits of pollination to crops are often enhanced under effective pest control [6, 7], while the use of certain pesticides has shown harmful effects on pollinators reducing the final yield in the short and long term [8, 9]. In this context, growers face a dilemma when choosing products or practices for pest management because the most effective pest control methods can also be detrimental to beneficial insects, such as bees.

Neonicotinoids are widely used systemic insecticides that are well recognized for their broad-spectrum activity against sap-sucking and chewing pests [10], and for their negative effects on bees [11], such as reduced longevity [12], physiological alterations of glandular development [13], altered foraging behavior [14], and reduced colony growth [15]. Because neonicotinoids are absorbed by all parts of the plant, they can be applied in many ways, such as foliar spray, seed dressing, soil treatment, seedling dipping, and trunk injections [16], but prophylactic seed and soil treatments are the most common application methods [10]. Once applied, neonicotinoids exhibit systemic uptake and are translocated to different plant tissues providing pest protection for some weeks. However, neonicotinoids can also accumulate in soils, nectar, and pollen which increases exposure to non-target organisms, including insect pollinators [17, 18]. Despite the challenge that this situation represents for sustainable agriculture, there is little information to assess how different pesticide application methods impact both pest control and pesticide residues that could harm pollinators [19].

Squash crops *(Cucurbita pepo)*, as well as other monoecious cucurbits, are entirely dependent on bee pollination to produce fruits [20]. The most common and efficient bee species pollinating cucurbits in eastern North America are *Apis mellifera*, *Bombus impatiens*, and the solitary ground-nesting bee *Eucera (Peponapis) pruinosa* [21, 22]. *E*. *pruinosa*, also known as squash bees, rely mostly upon pollen of Cucurbitaceae to rear offspring, and females commonly nest within the cultivated fields [23], which means that these bees can spend their entire life cycle proximate to and feeding on Cucurbita crops, increasing the risk of pesticide exposure [24]. For instance, in hoop houses with squash plants treated with systemic insecticides, imidacloprid soil applications greatly affected squash bees by reducing 85% of the nests initiated and 89% of the offspring [25].

The striped cucumber beetle, *Acalymma vittatum* (F.) (Coleoptera: Chrysomelidae), is the major insect pest of squash crops in the northeastern and midwestern United States and eastern Canada [26]. Adult beetles feed on cotyledons, foliage, flowers, and fruits, and larvae feed exclusively on roots [27, 28]. Early season damage and the transmission of bacterial wilt (*Erwinia tracheiphila*) are the biggest concerns associated with the striped cucumber beetle [29, 30]. Previous work has demonstrated that squash cultivars vary in their attractiveness to the striped cucumber beetle, with *C*. *pepo* subsp. *pepo* cultivars being more severely damaged than *C*. *pepo* subsp. *Texana* [27].

Organophosphates, carbamates, pyrethroids, and neonicotinoids are often used to manage striped cucumber beetle throughout the growing cycle, but neonicotinoids are commonly applied as prophylactic measures at planting through seed treatments, soil drenches, or early foliar applications to provide an initial protection window [29, 31, 32]. About 173,000 acres of cucurbits are treated with neonicotinoids every year in the United States [33]. Thiamethoxam, a second-generation neonicotinoid, has been registered for in-furrow soil treatments and foliar sprays for several years. In 2009, it was also registered as the insecticidal component of seed treatments (Cruiser, as a component in FarMore FI400), becoming the largest agricultural use for thiamethoxam [34, 35]. On average, 800,000 lbs. a.i. of thiamethoxam are annually used for seed treatments on various field crops [36]. Although neonicotinoid residues have previously been found in pollen, nectar, and soil of Cucurbita crops [17, 19, 24], the effect that different application methods have on the magnitude of these residues and on their pest control efficiency remains to be tested.

The aim of this study was to evaluate how different prophylactic thiamethoxam application methods (seed treatment, in-furrow application, and early foliar spray) and two different plant varieties impact pest control, bee visitation, yield, and pesticides residues in pollen and nectar of squash crops. Our goal was to elucidate whether there is a solution to the growers’ dilemma between pest control and pollinator protection by using a particular thiamethoxam application method and a less attractive variety to the striped cucumber beetle that would reduce pest pressure and minimize pesticide exposure to bees.

## 2. Materials and methods

### Field experimental design

We conducted a field experiment at the Homer C. Thompson Vegetable Research Farm in Freeville, New York, in 2019. We used two cucurbit cultivars that differ in their attractiveness to striped cucumber beetles: Golden Zucchini (*C*. *pepo subsp*. *pepo*) as a highly preferred cultivar and Success PM straightneck summer squash (*C*. *pepo subsp*. *texana*), a non-preferred cultivar to the beetle [27]. We obtained certified organic seeds that were all treated with fungicides at the label rate to avoid early damage by pathogens with the following products: Mefenoxam (Apron XL, 33.3% active ingredient (a.i.), 0.42g/kg of seed, by Syngenta), Fludioxonil (Maxim 4FS, 40.3% a.i., 0.1g/kg of seed, by Syngenta), and Azoxystrobin (Dynasty, 9.6% a.i., 0.25g/kg of seed, by Syngenta).

We then tested the following four thiamethoxam application methods at the high commercial label rates (Table 1): (1) In-furrow application after sowing (2) Foliar spray application three weeks after sowing when the plants had three true leaves, (3) Seed treatment, and (4) No insecticides applied. For the fungicides and the thiamethoxam seed treatments we used a laboratory-scale rotary pan coater, R-6 (Universal Coating Systems, Independence, OR, USA) in the third author’s Seed Technology lab at Cornell Agri-Tech for all seed treatment applications. The general methodology used for seed treatment application as a seed dressing is described by Afzal *et al*., (2020) [37]. The experimental field did not receive neonicotinoid applications for at least the last three years and it was previously cultivated with kale, sorghum, and rye. (Personal communication with Steven P. McKay, farm manager). Surrounding crop fields with tomato and corn were at least 10 meters apart from the experiment.

**Table 1:** Commercial products, product rates, and active ingredient rates used in the different thiamethoxam application methods tested.

	Thiamethoxam application method		
	In-furrow	Foliar spray	Seed coating
**Commercial product (% of thiamethoxam)**	Platinum 75 SG (75%) Syngenta	Actara (25%) Syngenta	Cruiser 5 FS (47.6%) Syngenta
**Application rate of commercial product (g/ha)**	257.1	385.2	17.1
**Active ingredient applied per hectare (g/ha)**	192.82	96.3	8.1
**Active ingredient applied per plant or seed (g)**	0.028 g/plant	0.014 g/plant	0.00125 g/seed
(Plant density 6858 plants/ha)			

We applied all the treatments in a seven-fold replicated randomized block design with a total of 56 plots (4 treatments x 2 varieties x 7 replicates). Each plot consisted of four 4.8 m long rows, containing 6 plants each. The plants were spaced 0.6 m apart within the rows and 2.43 m among rows, for a total of 24 plants per plot. Plots were spaced 5 m apart to avoid potential pesticide contamination between treatments. Fertilizer (NPK 13-13-13) was applied at a rate of 40 Kg/h. All the rows had a drip irrigation system installed and were covered with black plastic mulch. On June 3rd and 4^th^, all the plots were direct-seeded by hand with two seeds per hole. After a week, if both seeds germinated, the shortest seedling was removed to leave only one plant per hole.

#### Herbivory and bacterial wilt incidence

A trained observer estimated herbivory on eight plants growing in the two central rows of each plot by visually assessing the defoliation percentage of the four youngest leaves. In addition, we counted the number of herbivores on the plant and within 10 cm around each plant. Every plot was sampled weekly for four weeks from June 27th to July 15^th^, when all the plots started to flower, and the striped cucumber beetle population decreased and moved to the flowers for mating aggregation [26]. On June 16^th^, we also recorded the number of dead plants per plot with leaves or stems wilted as a measure of bacterial wilt incidence [30].

#### Nectar and pollen collection

To determine how our thiamethoxam treatments impacted pesticide concentration in nectar and pollen, we collected nectar, and pollen samples from flowers in each plot, except for four central flowers in plants dedicated to measure yield. For nectar, muslin bags were placed over flower buds the day before anthesis to protect them from early visitors. The next day, the flowers were dissected by removing the calyx and the corolla to expose the filament and the receptacle base. Nectar was collected using a P200 micropipette, using a new disposable tip in every plot. We extracted the nectar from multiple flowers until at least 1.0 mL had been collected from each treatment plot. For pollen collection, we brought the anthers to the lab and using forceps, we removed the pollen to collect at least 100mg of pollen per plot. Forceps were washed with alcohol (96%) and gloves were changed between samples to avoid cross contamination. Nectar and pollen samples were frozen at -20C until extraction for pesticide analysis. To distinguish if sampling time changes the probability to find pesticide residues, we collected weekly samples in July “early flowering” and August “late flowering”.

#### Bee visitation

On sunny mornings (7:00 am to 11:00 am) during the flowering period, two trained observers walked the plots, starting at opposite ends of the field, to record the number of flowers and the identity and number of bees visiting the flowers at each plot for 4 min. Every plot was sampled twice per week for four weeks from July 27th to August 15th.

#### Harvest

To calculate the fruit number and the fruit weight per plot we harvested all the fruits from four labeled plants from the two internal rows in every plot. We counted and weighted the fruits every week from July 15^th^ to August 23^rd^. We estimated total yield by adding the weight of all fruits per plant during that period.

### Pesticide analysis

The nectar, and pollen samples collected were extracted and analyzed at the Cornell Chemical Ecology Core Facility to quantify pesticide residues. Samples were screened for thiamethoxam and clothianidin by liquid chromatography mass spectrometry (LC-MS/MS). We screened for clothianidin given that it is the main metabolite of thiamethoxam in plants and insects [38] (See supplementary information for detailed methodology on the extraction and liquid chromatography and mass spectrometry conditions, S1 Table). We also screened for metalaxyl, azoxystrobin, and fludioxonil, because these fungicides were used in all the treatments as seed dressings to avoid pathogens (S2 Table).

#### Pesticide hazard quotient (HQ)

Based on the thiamethoxam and clothianidin residues found in pollen and nectar, we estimated the pesticide HQ for the squash bee *Eucera (Peponapis) pruinosa*, given that this species consumes mostly pollen and nectar of Cucurbit plants [39], and is considered one of the most important pollinators of squash crops in North America [22, 40, 41]. We calculated the HQs based on the following formula [24]:

$$HQ=\frac{\left(Mean\;concentration\;quantified\;residues\;in\;matrix)(Amount\;of\;exposure\;to\;matrix\right)}{Honey\;bee\;Oral\;or\;contact\;LD50}$$

The concentrations of the residues in pollen and nectar are reported as nanograms of active ingredient per gram (ng a.i./g), the amount of exposure to the matrix (nectar or pollen) in g/bee, and the LD50s in ng a.i./bee (LD50: Lethal doses causing 50% mortality). The amount of exposure to nectar and pollen for *E*. *(Peponapis) pruinosa* was obtained from previous calculations made by Willis Chan *et al*. (2019) [24]. The estimated amount of pollen consumed by a larva is 0.0542g pollen/nest cell. For adult females, contact exposure due to manipulation of pollen is estimated as five times the amount of pollen consumed by each larva given that adult females provision five cells on average (5*0.0542g pollen/nest cell). For adult consumption, a female squash bee can consume 790 mg of Cucurbita nectar and there is no estimate available for the amounts of pollen it can consume. We obtained honey bee LD50s values from the ECOTOX database of the US-Environment Protection Agency (http://cfpub.epa.gov/ecotox/). We added all the HQs calculated for the different stages and matrices of exposure to determine a combined HQ per thiamethoxam treatment and sampling period. An HQ≥1 indicates these levels of pesticide exposure can cause ≥50% mortality, constituting a lethal hazard.

### Statistical analysis

We performed all the analyzes in R version 3.3.0 [42]. To evaluate the effects of plant variety, thiamethoxam application methods, and their interaction on the percentage defoliation and the number of plant death with bacterial wilt symptoms we used generalized linear models (GLM), with quasibinomial error distribution and logit link for defoliation and poisson distribution and logit link for plant death. For the effects of plant variety, thiamethoxam application methods, and their interaction on fruit number and fruit weight we used linear models, with fruit number and fruit weight square rooted to meet normality assumptions. To evaluate the effects of plant variety, thiamethoxam application methods, and their interaction on bee visits per plot we used GLM, with quasipoisson error distribution and log link. Finally, to assess the effects of plant variety, thiamethoxam application methods, and their interaction on the thiamethoxam concentration residues in pollen and nectar we used GLM, with gamma error distribution and inverse link. To assess the statistical significance of each explanatory variable we used the Chi-square test for the GLM models and F-test for the linear models. We also performed Tukey HSD post-hoc tests with the function *HSD*.*test* of the package *agricolae* [43] to evaluate the differences among thiamethoxam application methods or plant varieties.

In order to evaluate the direct and indirect effects of thiamethoxam residues on yield through defoliation and bee visits, we conducted a path analysis using the *piecewiseSEM* package [44]. We hypothesized that thiamethoxam concentration in floral rewards, as an estimation of the residues in all the plant tissues, would have a negative impact on percent defoliation and bee visits. We also hypothesized that percentage defoliation would have a negative impact on yield and bee visits, given a decrease in the number of flowers, and that bee visits would have a positive impact on yield. In the path models, yield was square root transformed to normalize model residuals, bee visits were fitted with Poisson error distribution and log link, and defoliation was fitted with quasibinomial error distribution and logit link. The overall fit of the path model was tested using Shipley’s d-separation test for each possible independent claim, and Fisher’s C statistics to test whether observed levels of correlation across all independent claims can be explained by random variation. Regression coefficients presented are unstandardized. To improve model fit, we modified our models using a backward and forward stepwise process based on Akaike’s information criterion (AIC) where nonsignificant relationships were removed (pathways where P > 0.05), and significant relationships were added, and AIC values reassessed. Models with lower AIC values are considered to be better-fitted models [45].

## 3. Results

### Effect of thiamethoxam application method and plant variety on herbivory and bacterial wilt

The striped cucumber beetle (*A*. *vittatum*) was the most common herbivore in our study with 96.4% of the individuals observed. We also found a few squash bugs (3.2% *Anasa tristis*) and spotted cucumber beetles (0.4% *Diabrotica undecimpunctata*). The percentage defoliation varied from 0 to 50% on average per plot. As the percentage defoliation increased, fruit number and fruit weight significantly decreased, showing the detrimental impact of the striped cucumber beetle on yield (S1 Fig).

**Fig 1:**
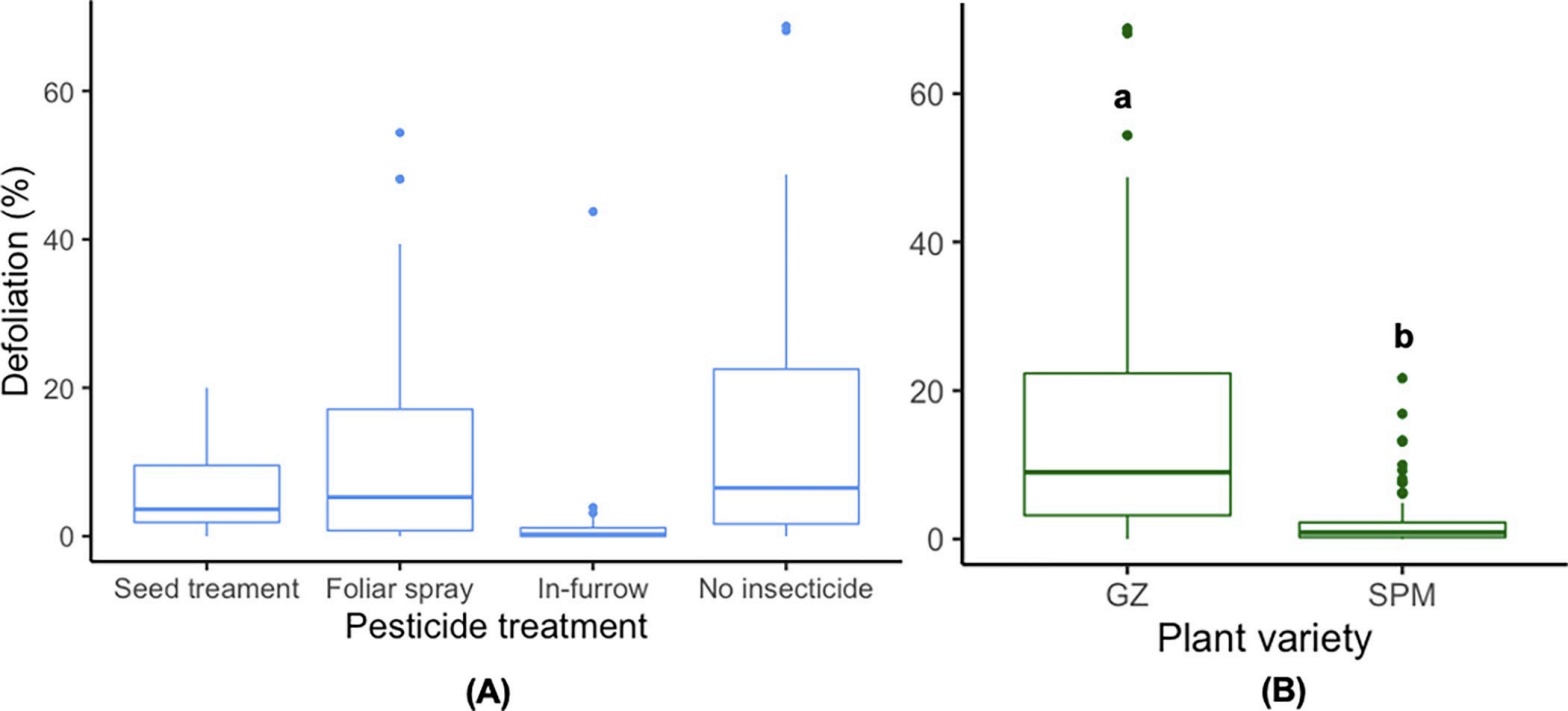
**A.** Percentage defoliation in squash plants treated with different thiamethoxam application methods. **B.** Percentage defoliation in two plant varieties of squash crops, GZ: Golden Zucchini, SPM: Success PM. Whiskers indicate the range of the defoliation found, excluding outliers. Upper, middle, and lower quartiles indicate the greatest, average, and lowest defoliation percentage estimated, respectively. Different letters indicate significant differences between levels of the treatment based on Tukey’s HSD post-hoc test (p < 0.05).

We found that both thiamethoxam application method and plant variety but not the interaction, had significant effects on the defoliation percentage (Table 2). Among the thiamethoxam application methods, in-furrow applications showed the lowest defoliation in comparison to the rest of the treatments (Fig 1A), although the difference was not significant in the post-hoc Tukey test. For plant varieties, Golden Zucchini showed higher average percentage defoliation (15.4% +/- 13.9_SD_) in comparison to Success PM (8.3% +/- 13.4 _SD_) (Fig 1B).

**Table 2:** ANOVA results for the effect of thiamethoxam application method, plant variety, and their interactions on percentage defoliation, number of dead plants with bacterial wilt symptoms, fruit number, fruit weight, bee visits per plot, and bee visits per flower in squash plants.

Response variable				
Defoliation	*df*	*X2*	*p value*	
Thiamethoxam application method	3, 161	9.62	<0.0001	***
Plant variety	1, 164	7.12	<0.0002	***
Thiamethoxam application method x Plant variety	3, 158	0.69	0.08	
**Dead plants with bacterial wilt symptoms**	***df***	***X2***	***p value***	
Thiamethoxam application method	3, 52	58.1	<0.0001	***
Plant variety	1, 51	9.29	<0.002	**
Thiamethoxam application method x Plant variety	3, 48	17.5	0.0005	***
**Fruit number per plant**	***df***	***F***	***p value***	
Thiamethoxam application method	3, 151	16.3	<0.0001	***
Plant variety	1, 150	1.45	0.229	
Thiamethoxam application method x Plant variety	3, 147	1.86	0.137	
**Fruit weight per plant**	***df***	***F***	***p value***	
Thiamethoxam application method	3, 151	29.98	<0.0001	***
Plant variety	1, 150	2.47	0.11	
Thiamethoxam application method x Plant variety	3, 147	2.42	0.06	
**Bee visits per plot**	***df***	***X2***	***p value***	
Thiamethoxam application method	3, 164	116.52	<0.0001	***
Plant variety	1, 163	10.9	0.022	*
Thiamethoxam application method x Plant variety	3, 160	18.08	0.033	*
**Bee visits per flower**	***df***	***X2***	***p value***	
Thiamethoxam application method	3, 164	9.25	<0.0001	***
Plant variety	1, 163	0.18	0.389	
Thiamethoxam application method x Plant variety	3, 160	1.09	0.22	

Stars demark the significance level (*P < 0.05, **P < 0.01, ***P < 0.001).

**Fig 2:**
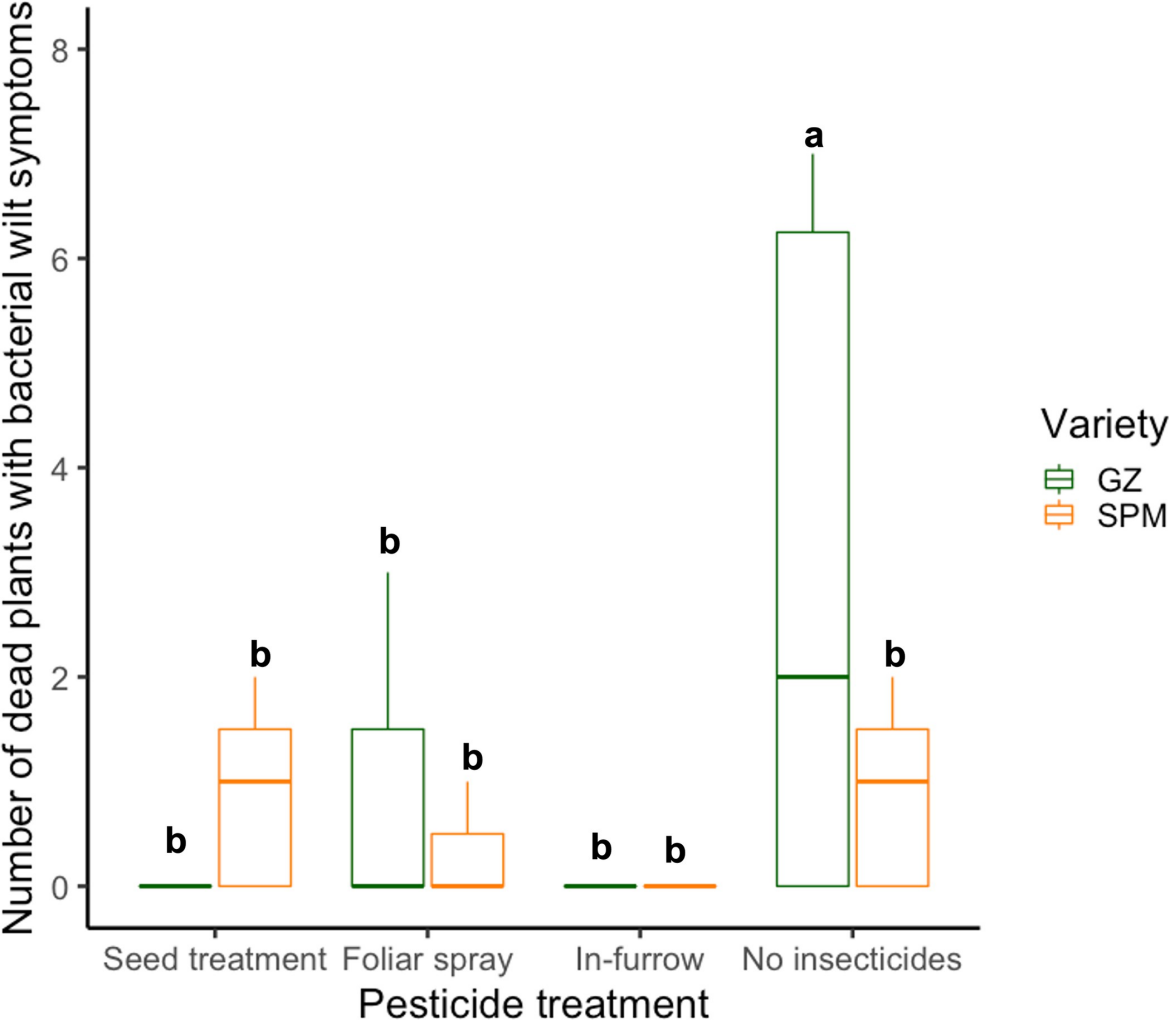
Number of dead plants per plot with bacterial wilt symptoms treated with different thiamethoxam application methods from two different plant varieties: GZ: Golden Zucchini, SPM: Success PM. Whiskers indicate the range of the numbers found, excluding outliers. Upper, middle, and lower quartiles indicate the greatest, average, and lowest number of dead plants, respectively. Different letters indicate significant differences between levels of the treatment based on Tukey’s HSD post-hoc test (p < 0.05).

The number of dead plants with bacterial wilt symptoms was significantly affected by the thiamethoxam application method, the plant variety, and the interaction of both variables (Table 2). Based on the Tukey’s HSD post-hoc test the combination of no insecticides and the plant variety Golden Zuchinni showed the highest number of dead plants compared to the rest of the treatments with no other differences among them (Fig 2).

### Effect of thiamethoxam application method and plant variety on yield

**Fig 3:**
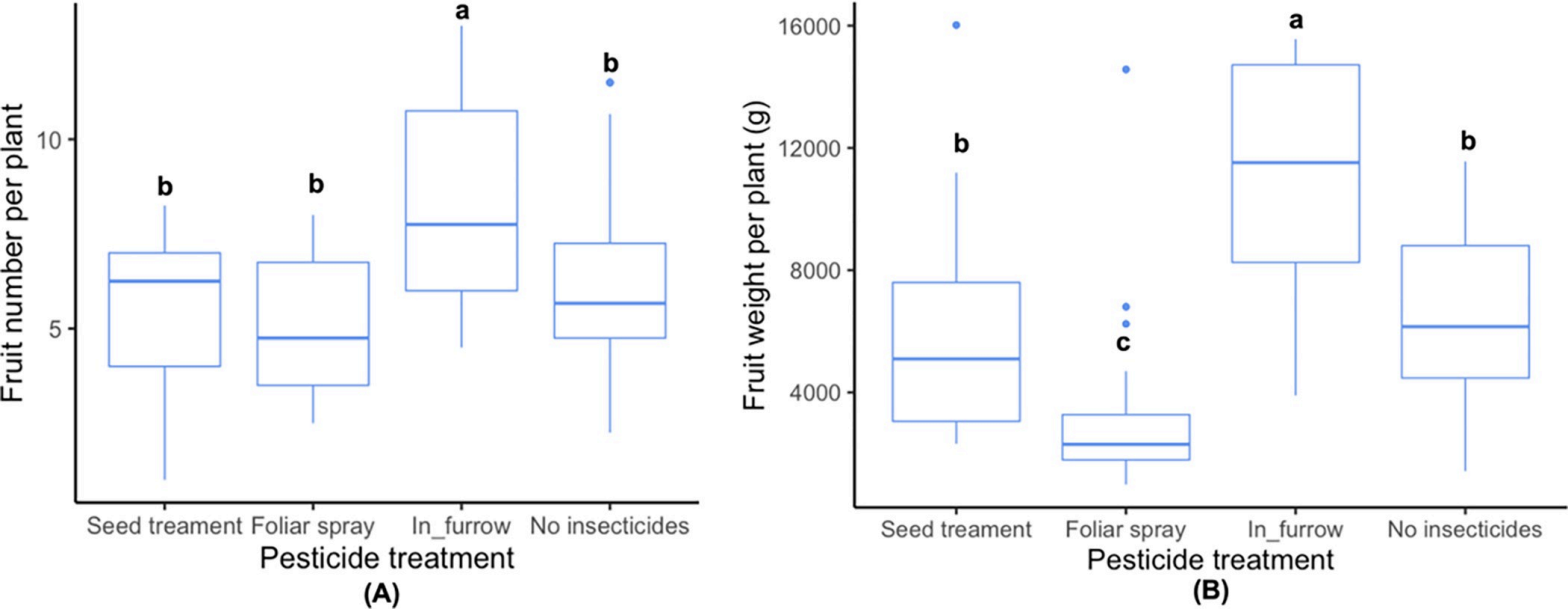
Effect of different thiamethoxam application methods on **(A)** fruit number and **(B)** fruit weight per plant in squash crops. Whiskers indicate the range of numbers and weights of fruits harvested, excluding outliers. Upper, middle, and lower quartiles indicate the greatest, average, and lowest fruit numbers and fruits weights quantified, respectively. Different letters indicate significant differences between levels of the treatment based on Tukey’s HSD post-hoc test (p < 0.05).

We found that the thiamethoxam application method but not variety had a significant effect on fruit number and fruit weight (Table 2). Based on post-hoc pairwise contrasts, plants treated with in-furrow applications produced the highest fruit number and fruit weight in comparison to all other application methods (Fig 3A and 3B).

### Effect of thiamethoxam application method and plant variety on bees

*Eucera (Peponapis) pruinosa* was the most common flower visitor of the crop with 57% of the recorded visits, followed by *Bombus impatiens* with 25.8%, and *Apis mellifera* with 14.9%. We also recorded a few visits from bees in the genera *Augochlorella*, *Lasioglossum*, *Andrena*, and *Melissodes*. As the number of bee visits per plot increased, fruit number and fruit weight per plant significantly increased (S1 Fig).

**Fig 4:**
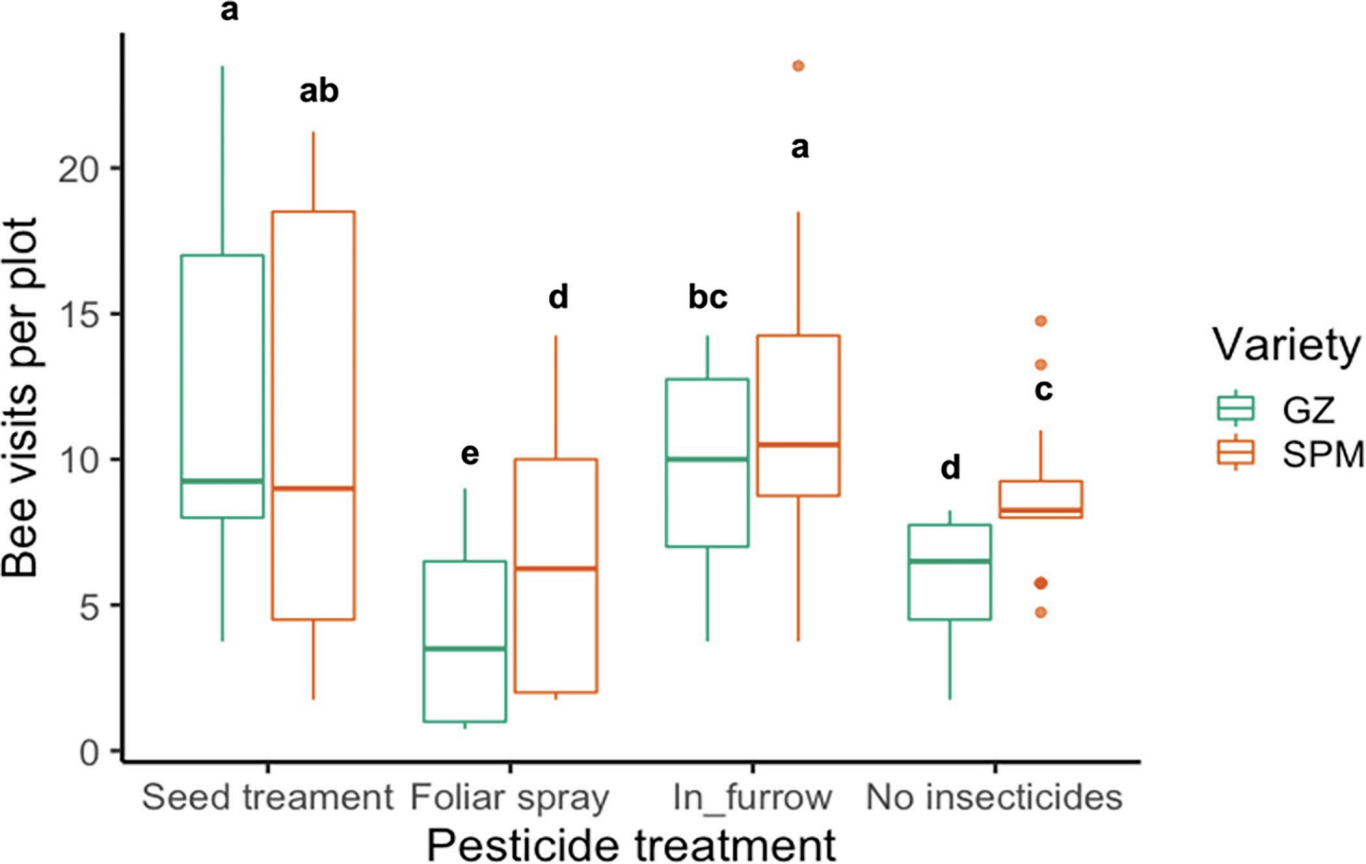
Bee visits per plot in two varieties of squash crops (GZ: Golden Zucchini and SPM: Success PM) treated with different thiamethoxam application methods. Whiskers indicate the range of bee visits counted, excluding outliers. Upper, middle, and lower quartiles indicate the greatest, average, and lowest number of bee visits, respectively. Different letters indicate significant differences between levels of the treatment based on Tukey’s HSD post-hoc test (p < 0.05).

We found that thiamethoxam application method, plant variety, and their interaction had significant effects on the number of bee visits per plot (Table 2). The seed treatment for both varieties and the in-furrow treatment for Success PM received the highest number of bee visits per plot while Golden Zucchini treated with foliar spray received the lowest (Fig 4).

#### Pesticide residues found in nectar and pollen

In nectar samples, thiamethoxam was most frequently detected in plants treated with in-furrow applications, followed by seed treatment. In-furrow application was also the treatment with the highest thiamethoxam mean concentration in nectar with 6.58 ppb. For pollen, thiamethoxam was detected in 100% of the samples coming from in-furrow applications and it was also the treatment with the highest mean concentration in pollen with 13.8 ppb, followed by 4.42 ppb in the seed treatment. Unexpectedly, we found thiamethoxam residues in pollen samples from the no insecticide treatment, even though no neonicotinoid applications have been made in this lot in the last three years. Clothianidin, the main thiamethoxam metabolite, was only found and quantified in in-furrow applications. Table 3 shows the frequency of pesticide detection and the mean concentration of thiamethoxam and clothianidin residues found in nectar and pollen samples for the different thiamethoxam application methods.

**Table 3:** Thiamethoxam and clothianidin residues found in nectar and pollen from early flowering (July) and late flowering (August) in squash plants treated with different thiamethoxam application methods.

	No insecticide		Foliar spray		In-furrow		Seed treatment	
	Early	Late	Early	Late	Early	Late	Early	Late
**Thiamethoxam in nectar** (LOD 0.05 ppb, LOQ 0.15 ppb)	*N = 13*	*N = 6*	*N = 14*	*N = 7*	*N = 16*	*N = 13*	*N = 17*	*N = 9*
Frequency of detection (%)	0%	0%	21.40%	14%	87.50%	92.30%	67.70%	11.10%
Frequency of quantification (%)	0%	0%	0%	0%	87.50%	92.30%	17.60%	0%
Range ppb*					1.13–32.08	0.28–2.3	0.33–0.51	
mean +/- SD ppb*					6.58+/-8.91	1.48+/-0.63	0.41+/-0.09	
**Thiamethoxam in pollen** (LOD 0.056 ppb, LOQ 0.168 ppb)	*N = 8*	*N = 6*	*N = 12*	*N = 5*	*N = 10*	*N = 9*	*N = 14*	*N = 5*
Frequency of detection (%)	75%	17%	83.30%	40%	100%	100%	78.60%	20%
Frequency of quantification (%)	50%	0%	33.30%	0%	100%	100%	28.60%	20%
Range ppb*	0.21–0.64		0.31–1.13		4.87–41.2	0.67–8.84	0.3–1.9	4.42
mean +/- SD ppb*	0.37+/-0.19		0.65+/-0.37		13.8+/-12.6	3.91+/-2.81	1.11+/-0.67	4.42+/-0
**Clothianidin in nectar** (LOD 0.1 ppb, LOQ 0.3 ppb)	*N = 13*	*N = 6*	*N = 14*	*N = 7*	*N = 16*	*N = 13*	*N = 17*	*N = 9*
Frequency of detection (%)	0%	0%	0%	0%	31.20%	0%	0%	0%
Frequency of quantification (%)	0%	0%	0%	0%	18.75%	0%	0%	0%
Range ppb*					0.32–1.1			
mean +/- SD ppb*					0.79+/-0.41			
**Clothianidin in pollen** (LOD 0.14 ppb, LOQ 0.42 ppb)	*N = 8*	*N = 6*	*N = 12*	*N = 5*	*N = 10*	*N = 9*	*N = 14*	*N = 5*
Frequency of detection (%)	0%	0%	0%	0%	100%	0.67%	0%	20%
Frequency of quantification (%)	0%	0%	0%	0%	100%	11%	0%	0%
Range ppb*					0.48–4.4	1.96		
mean +/- SD ppb*					1.79+/-1.7	1.96+/-0		

*Range and mean were calculated from the quantified samples

N: Number of samples

LOD: Limit of detection

LOQ: Limit of quantification

#### Effect of thiamethoxam application method and plant variety on the thiamethoxam concentration residues in flowers

**Fig 5:**
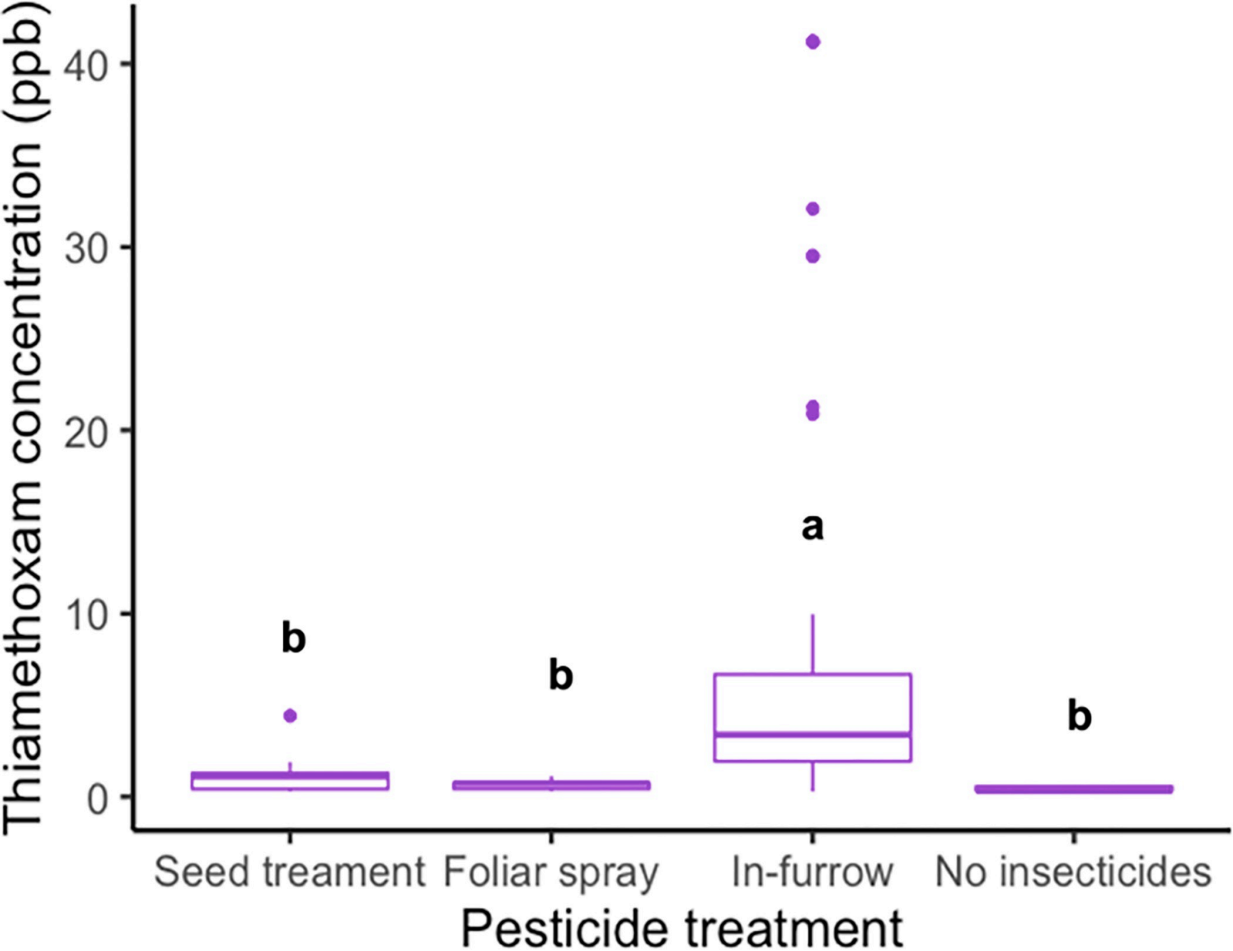
Thiamethoxam concentrations of the residues quantified (ppb) in pollen and nectar samples of squash plants treated with different thiamethoxam application methods. Whiskers indicate the range of thiamethoxam concentrations found, excluding outliers. Upper, middle, and lower quartiles indicate the greatest, average and lowest thiamethoxam concentrations quantified, respectively. Different letters indicate significant differences between levels of the treatment based on Tukey’s HSD post-hoc test (p < 0.05).

The application method was a significant predictor of the thiamethoxam concentration residues quantified in flowers *(X*^*2*^_*(3*, *74)*_ *= 59*.*33*, *p-value <0*.*0001)*, where in-furrow applications resulted in the highest concentrations (Fig 5). There were not significant differences in thiamethoxam concentrations among plant varieties *(X*^*2*^_*(1*, *73)*_ *= 1*.*004*, *p-value = 0*.*436)* or a significant effect of the interaction between the application method and the plant varieties *(X*^*2*^_*(2*,*71)*_ *= 1*.*116*, *p-value = 0*.*714)*.

### Effects of thiamethoxam residues on yield through defoliation and bee visits

**Fig 6:**
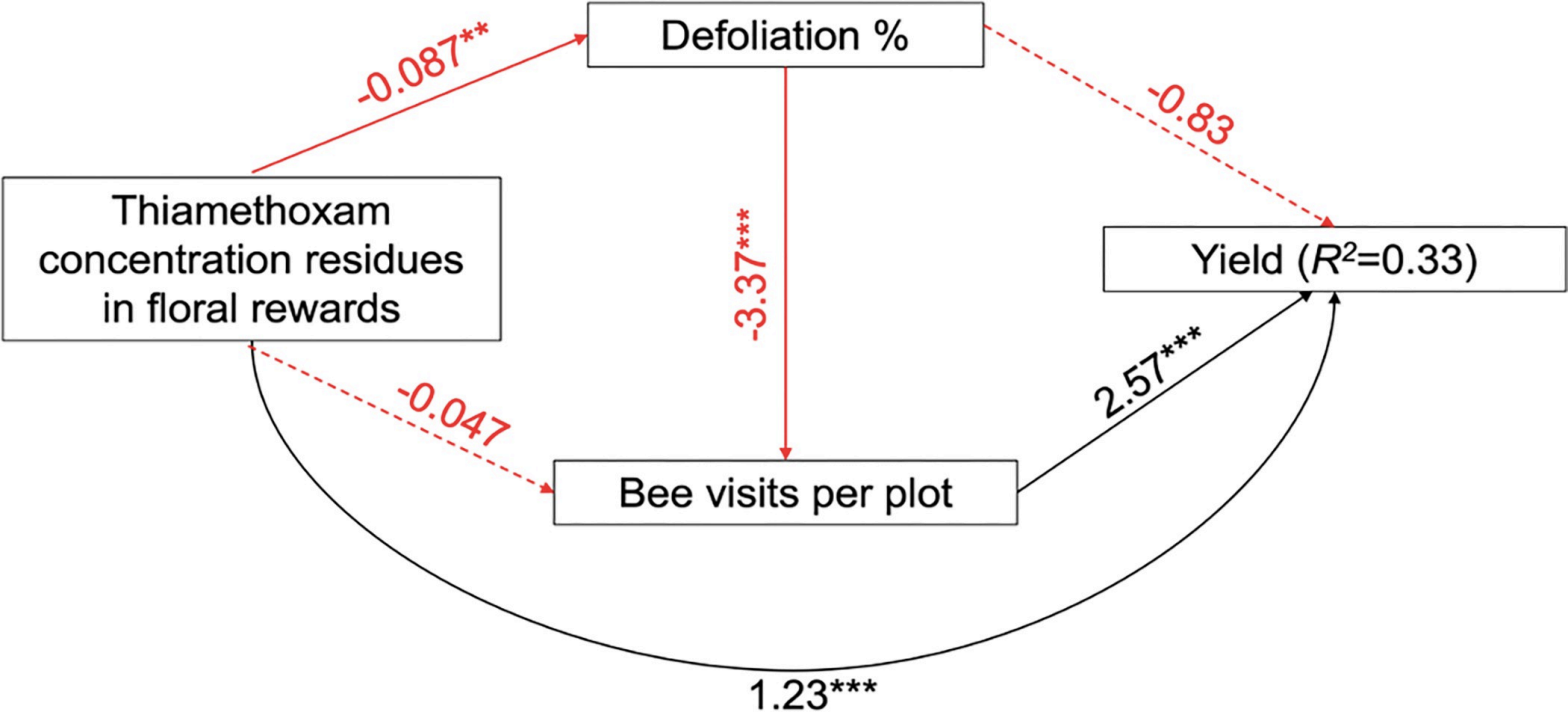
Path model for relationships between thiamethoxam concentration residues in pollen and nectar, defoliation, bee visits, and crop yield (Fisher’s C = 0.059, df = 2, P = 0.971) in both squash varieties. Solid lines indicate significant effects, while dashed lines denote nonsignificant effects. Black lines are positive effects, while red lines are negative effects. The number along the arrows are unstandardized path coefficients obtained from effect models, and stars demark the significance level (*P < 0.05, **P < 0.01, ***P < 0.001).

According to the best path model (Fig 6), we found that thiamethoxam had a direct and an indirect positive effect on yield. The indirect positive effect on yield was mediated by defoliation and bee visitation. As the concentration of thiamethoxam increased, the proportion of defoliation in squash plants decreased, which led to an increase in bee visitation and a subsequent increase in yield. The thiamethoxam concentration residues had no significant effect on bee visits, and defoliation had no significant direct effect on yield.

#### Hazard quotients calculation for *Eucera (Peponapis) pruinose*

Hazard quotients (HQ) for thiamethoxam and clothianidin residues in pollen and nectar for larvae and adult females of squash bees are presented in Table 4. The HQ for in-furrow applications in early flowering was the highest (HQ_Thiamethoxam_ = 1.326, HQ_Thiamethoxam+Clothianidin_ = 1.542) and the only above 1, representing a potential lethal hazard for squash bees.

**Table 4:** Hazard quotients (HQ) estimated for *Eucera (Peponapis) pruinosa* with potential exposure to nectar and pollen from flowers produced early (July) and late (August) in the flowering period of squash plants treated with different thiamethoxam application methods.

			Pollen			Nectar		
		Thiamethoxam treatment	Mean conc. in matrix (MC)	HQ Larvae (Oral)	HQ Adult female (Contact)	Mean conc. in matrix (MC)	HQ Adult female (Oral)	Combined HQ
			ng/g or ppb	0.0542g/bee*MC/LD50	(5*0.0542g/bee*MC)/LD50	ng/g or ppb	(0.78g/bee*MC)/ LD50	∑HQ
**Thiamethoxam**^**1**^: Oral LD50 (5 ng a.i./bee), Contact LD50 (25 ng a.i./bee)	**Early flowering sampling**	No insecticide	0.37	0.004	0.004	ND	__	0.008
		Foliar spray	0.65	0.007	0.007	NQ	_	0.014
		In-furrow	13.8	0.150	0.150	6.580	1.026	**1.326**
		Seed coating	1.11	0.012	0.012	0.410	0.064	0.088
	**Late flowering sampling**	No insecticide	NQ	_	_	ND	_	_
		Foliar spray	NQ			NQ	_	_
		In-furrow	3.91	0.042	0.042	1.480	0.231	0.316
		Seed coating	4.42	0.048	0.048	NQ	_	0.096
**Clothianidin 1:** Oral LD50 (3.5 ng a.i./bee), Contact LD50 (39 ng a.i./bee	**Early flowering sampling**	No insecticide	ND	___	_	ND	_	_
		Foliar spray	ND			ND	_	_
		In-furrow	1.79	0.028	0.012	0.79	0.176	0.216
		Seed coating	ND	_	_	ND	_	_
	**Late flowering sampling**	No insecticide	ND	_	_	ND	_	_
		Foliar spray	1.96	_	_	ND	_	_
		In-furrow	ND	_	_	ND	_	_
		Seed coating	NQ	_	_	ND	_	_

^1^ Insecticide toxicity data obtained from ECOTOX Database

ND: Not detected NQ: Not quantified.

Thiamethoxam

Limit of detection in nectar 0.05 ppb, limit of detection in pollen: 0.056 ppb

Limit of quantification in nectar 0.15 ppb, limit of quantification in pollen 0.168 ppb.

Clothianidin

Limit of detection in nectar 0.1 ppb, limit of detection in pollen 0.14 ppb

Limit of quantification in nectar 0.3 ppb, limit of quantification in pollen 0.42 ppb

## 4. Discussion

We found that in-furrow applications of thiamethoxam in squash crops showed the least defoliation and the highest fruit number and fruit weight compared to seed treatments and foliar spray. However, in-furrow is also the treatment that generated the most frequent and highest concentrations of thiamethoxam in pollen and nectar, reaching lethal levels for squash bees during the first month of flowering. These results can be explained by the higher amount of active ingredient used in in-furrow commercial applications compared to the others and a continued uptake of the insecticide from the soil by the plants [46, 47]. Seed treatment was the second-best treatment to avoid defoliation but there were no differences in fruit number or fruit weight in comparison with the foliar spray treatment or the control with no insecticides. This is a concerning result because seed treatments are commonly used by cucurbit growers [34], but their use does not always result in higher yields [48]. Foliar spray was also not different in the percentage defoliation and fruit number in comparison with the treatment with no insecticides, similar to what has been found in a previous study using imidacloprid with other cucurbits [29]. This may be caused by a short duration of the insecticide in the plant due to photodegradation or washed away by rain, which often leads to multiple applications in the field [49].

The highest number of dead plants with bacterial symptoms was found for the plots with no insecticides and the variety Golden Zucchini, with no other differences among the rest of the treatments. These results show that all thiamethoxam applications reduced bacterial wilt incidence but only for the variety with higher defoliation which can be a result of the lower incidence of the beetles or due to a reduced susceptibility of the variety [27, 30].

While plant varieties differed in their percentage of defoliation, we found no differences in fruit number or fruit weight, which could indicate a potential capacity of Golden Zucchini, the most defoliated variety, to compensate for the herbivory effect, as it was previously shown for *Cucumis sativus*, where damaged plants had a higher photosynthetic capacity [50].

We did not find differences in the concentration of pesticide residues in pollen and nectar between varieties, the opposite of what has been found in previous studies. For instance, in sunflower, different plant varieties treated with imidacloprid resulted in different levels of residues in flowers [51]. Also, in Japan, in soils contaminated with dieldrin and endrin, two pyrethroid insecticides, it was shown that cucurbits took up more pesticides than any other plant family tested, and there were significant differences in the concentrations found in different cultivars of *Cucurbita* [52]. This variation in pesticide translocation between varieties has been related to differences in the distribution and mass of the root systems [53].

We recorded a higher number of bee visits for the seed treatment and the in-furrow treatment compared to the foliar spray treatment and the control, as well as a higher number of visits to the Success PM variety compared to Golden Zucchini, which can be explained by the higher density of flowers in less defoliated plants [50] and the previous evidence that bees cannot taste neonicotinoids and even preferred solutions containing them [54]. While we found that in-furrow applications result in lethal hazard levels for squash bees in the first month of flowering, we are likely underestimating the risk to ground-nesting bees because our study did not include pesticide analysis of the soil [24], and squash bees are also exposed to contaminated soil by contact during nest building. Our study also supports previous results showing that in squash plants treated at planting with soil-applied imidacloprid, *E*. *pruinosa* populations declined, while seed treatments with thiamethoxam had no measurable impact on nesting or offspring produced [25].

We found higher thiamethoxam concentrations in pollen compared to nectar, the same as previously found in pumpkins treated with imidacloprid and thiamethoxam [19]. The increased concentration in pollen means that the pesticide exposure for bumble bees and honey bees is potentially lower compared to squash bees considering that these bees collect negligible amounts of cucurbit pollen [39]. By collecting nectar and pollen samples at two-time points, we showed that pesticide residues decreased with time since application and therefore the risk for bees also decreased. This temporal decrease must be taken into account when estimating pesticide hazards.

Although in the path analysis we found that thiamethoxam residues had an indirect positive impact on bee visits per plot through reduced defoliation, prolonged exposure to insecticides can cause a reduction in bee populations [55] and likely a reduction in crop yields in the long term. We also found a direct positive effect of thiamethoxam concentrations on the yield that was not explained by defoliation, which can be the result of the pest control effect of the insecticide on larvae of the striped cucumber beetle attacking the roots, which was not measured in this study. Root herbivory has been shown to not significantly decrease plant performance in *Cucurbita moschata* [56], but this has not been studied in *C*. *pepo*. While defoliation did not show a direct effect on yield in the path analysis, bee visits per plot did, highlighting once more the importance of bee pollination in squash. In cucumbers, for example, it was shown that insect pollination is the most important production driver far above fertilization, weed control, and pest control [57].

Finding pest control measures that maximize production without affecting pollinators is challenging, however, our results indicate that factors such as the initial amount of active ingredient applied and the time since application to flowering are worth evaluating in detail [58]. For instance, in sunflowers, the concentration of imidacloprid in capitulums has been shown to depend on the dose used in pesticide seed treatments, the residues being greater when the dose increased [59].

Much of the current research has focused either on pesticide effects on beneficial insects or pest control efficiency, however, it is important to integrate these two aspects of crop management to better assess the trade-off of pesticide use and to find solutions to reduce or avoid pesticide exposure to non-target organisms. In our study, we show that due to the lethal hazard for bees of thiamethoxam residues coming from in-furrow applications and the lack of pest control efficiency from the seed treatments and the foliar spray, commercial applications of thiamethoxam does not currently provide a sustainable solution for squash growers, representing a dilemma that requires further research on more efficient pesticide delivery methods as well as on non-pesticide pest control measurements. The incorporation of parasitoids [60] and entomopathogenic nematodes [61, 62] seems promising although they require broad development to increase efficiency and to facilitate the adoption by growers.

## Supporting information

S1 Fig Relation between the number of bee visits per plot and percentage defoliation on fruit number and fruit weight in squash plants. (DOCX)

S1 Table Pesticide analysis conditions: Retention times and optimized SRM acquisition parameters for pesticides. (DOCX)

S2 Table Metalaxyl, azoxystrobin, and fludioxonil residues found in pollen and nectar samples in squash plants treated with different thiamethoxam applications. (DOCX)

S1 File Supporting data for all the analysis. (XLS)
