# Supplementary material for: The pest control and pollinator protection dilemma: The case of thiamethoxam prophylactic applications in squash crops
Source: PLoS One. 2022 May 20;17(5):e0267984. doi: 10.1371/journal.pone.0267984 (PMC9122185; doi:10.1371/journal.pone.0267984)

**Electronic Supplementary information**

**S1 Figure.** Relation between the number of bee visits per plot and percentage defoliation on fruit number **(a, c)** and fruit weight **(b, d)** in squash plants. Dots represent the mean values per plot (N= 56 plots) and lines are the fixed‐effect predictions from the best model and the associated 95% confidence intervals (gray shaded area).


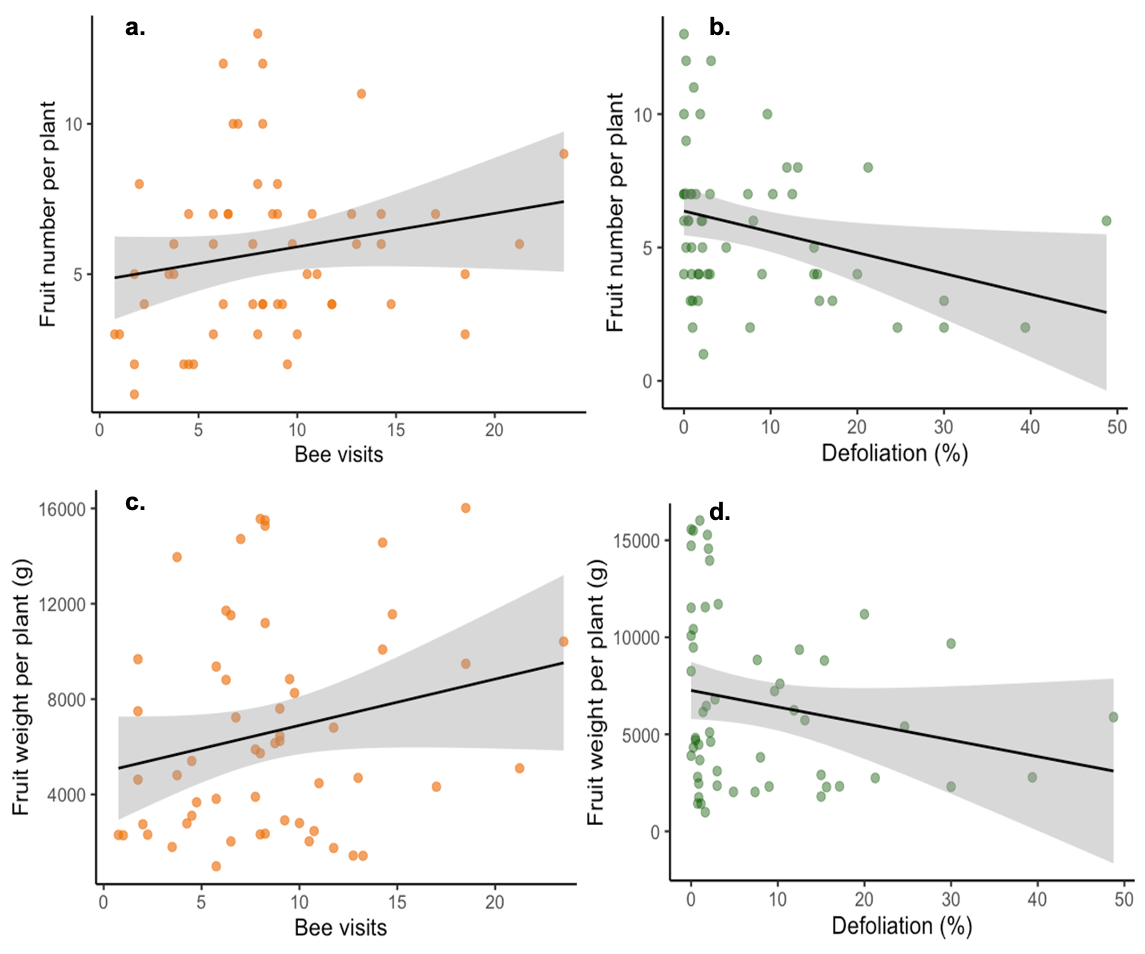

Supplement: S1 Fig — (DOCX) [file pone.0267984.s001.docx]
