# Supplementary material for: The pest control and pollinator protection dilemma: The case of thiamethoxam prophylactic applications in squash crops
Source: PLoS One. 2022 May 20;17(5):e0267984. doi: 10.1371/journal.pone.0267984 (PMC9122185; doi:10.1371/journal.pone.0267984)
Supplement: S1 Table — (DOCX) [file pone.0267984.s002.docx]

**Electronic Supplementary information**

**Pesticide analysis conditions:**

**Extraction:**

Pollen samples were extracted using a scaled-down version of the EN 15662 QuEChERS procedure as follows ^56^: Pollen samples (0.08‒0.5 g) were put into 2 mL screw cap tubes and mixed with 700 µL of acetonitrile and 200 µL of water. They were then homogenized for 1 min using ceramic beads (2.8 mm diameter) and a Bead Ruptor 24 (OMNI International, USA). After thorough homogenization, 330 mg of EN 15662 salts were added (203 mg MgSO4; 51 mg NaCl; 51 mg sodium citrate tribasic dihydrate; 25 mg sodium citrate dibasic sesquihydrate). Samples were vortexed then centrifuged at 7300 × g for 10 minutes. The supernatant was collected and transferred into a dispersive solid phase extraction (d-SPE) tube containing 150 mg MgSO_4_, 25 mg PSA and 25 mg C18EC. After the d-SPE step, 200 µL of supernatant were filtered (0.22 µm PTFE) and transferred into an HPLC vial for immediate analysis.

Nectar samples were centrifuged for 10 minutes at 7300 × g to remove solid particles. Subsequently, 100 µL of supernatant was diluted 1:1 with acetonitrile. The diluted nectar was then vortexed, filtered (0.22 µm PTFE) and transferred into an HPLC vial for immediate analysis.

**Liquid Chromatography and Mass Spectrometry:**

The analysis was performed with a Vanquish Flex UHPLC system (Dionex Softron GmbH, Germering, Germany) coupled with a TSQ Quantis mass spectrometer (Thermo Scientific, San Jose, CA). The UHPLC was fitted with an Acquity UPLC BEH C18 column (100 mm × 2.1 mm, 1.7 µm particle size). The mobile phase consisted of (A) Water containing 2 mM ammonium formate and 0.1% formic acid and (B) Acetonitrile/Water (98:2, v/v) with 2 mM ammonium formate and 0.1% formic acid. The temperature of the column was set at 40°C and the flow rate of the LC was 300 µL/min. The elution program was as follows: 1.5 min equilibration (0% B) prior to injection, 0‒0.5 min (0% B, isocratic), 0.5‒10 min (0%→70% B, linear gradient), 10‒112 min (70%→100% B, linear gradient), 12‒15 min (100% B, column wash), 15‒15.2 min (100%→2% B, linear gradient), 15.2‒17 min (2% B, re-equilibration). The flow from the LC was directed to the mass spectrometer through a Heated Electrospray probe (H-ESI). The settings of the H-ESI were spray voltage 2000 V for positive mode and 2000 V for negative mode, Sheath gas 55 (arbitrary unit), Auxiliary gas 25 (arbitrary unit), Sweep gas 1 (arbitrary unit), Ion transfer tube temperature 325°C, Vaporizer temperature 350°C. The MS/MS detection was carried out using the Selected Reaction Monitoring (SRM) mode. Two transitions were monitored for each compound: one for quantification and the other for confirmation. The SRM parameters for each individual compound are summarized in Table S1. The resolution of both Q1 and Q3 was set at 0.7 FWHM, the cycle time was 0.4 s and the pressure of the collision gas (argon) was set at 2 mTorr.

**Chemicals and Reagents:**

Acetonitrile and water of HPLC grade were purchased from EMD Millipore. LC-MS grade formic acid was purchased from Thermo Scientific. The 5M ammonium formate solution, the QuEChERS extraction packets (4 g MgSO4; 1 g NaCl; 1 g sodium citrate tribasic dihydrate; 0.5 g sodium citrate dibasic sesquihydrate) and the d-SPE kits (150 mg MgSO4, 25 mg PSA and 25 C18EC) were purchased from Agilent Technologies. Analytical standards of atrazine, clothianidin, thiamethoxam, azoxystrobin, metalaxyl and fludioxoinil were purchased from Millipore Sigma.

**S1 Table.** Retention times and optimized SRM acquisition parameters for pesticides (RT: Retention time, CE: Collision Energy).

| **Compound** | **RT (min)** | **RF Lens (V)** | **Polarity** | **Precursor (*m/z*)** | **Quantifying ion (*m/z*)** | **CE 1 (V)** | **Confirming ion (*m/z*)** | **CE 2 (V)** |
| --- | --- | --- | --- | --- | --- | --- | --- | --- |
| Thiamethoxam | 5.02 | 87 | Positive | 292 | 211.1 | 10 | 181 | 22 |
| Clothianidin | 5.48 | 78 | Positive | 250 | 169 | 13 | 131.9 | 17 |
| Atrazine | 8.14 | 129 | Positive | 216 | 174 | 18 | 104 | 29 |
| Metalaxyl | 8.36 | 104 | Positive | 280.1 | 220.1 | 14 | 248.1 | 10 |
| Fludioxonil | 9.52 | 130 | Negative | 246.9 | 180 | 30 | 126 | 32 |
| Azoxystrobin | 9.73 | 127 | Positive | 404 | 372 | 15 | 329 | 31 |
