# Supplementary material for: The pest control and pollinator protection dilemma: The case of thiamethoxam prophylactic applications in squash crops
Source: PLoS One. 2022 May 20;17(5):e0267984. doi: 10.1371/journal.pone.0267984 (PMC9122185; doi:10.1371/journal.pone.0267984)
Supplement: S2 Table — (DOCX) [file pone.0267984.s003.docx]

**Electronic Supplementary information**

**S2 Table.** Metalaxyl, Azoxystrobin and fludioxonil residues found in pollen and nectar samples in squash plants treated with different thiamethoxam applications.

|  | **No insecticide** | | **Foliar spray** | | **In-furrow** | | **Seed coating** | |
| --- | --- | --- | --- | --- | --- | --- | --- | --- |
|  | **Early** | **Late** | **Early** | **Late** | **Early** | **Late** | **Early** | **Late** |
| **Metalaxyl in nectar**  (LOD 0.02 ppb, LOQ 0.06 ppb) | *N=13* | *N=6* | *N=14* | *N=7* | *N=16* | *N=13* | *N=17* | *N=9* |
| Frequency of detection (%) | 15% | 0% | 7% | 0% | 0% | 0% | 12% | 11% |
| Frequency of quantification (%) | 0% | 0% | 7% | 0% | 0% | 0% | 6% | 0% |
| Range ppb* |  |  | 0.07 |  |  |  | 0.08 |  |
| mean +/- SD ppb* |  |  | 0.07+/-0 |  |  |  | 0.08+/-0 |  |
| **Metalaxyl in pollen**  (LOD 0.014 ppb, LOQ 0.042 ppb) | *N=8* | *N=6* | *N=12* | *N=5* | *N=10* | *N=9* | *N=14* | *N=5* |
| Frequency of detection (%) | 0% | 0% | 0% | 0% | 0% | 0% | 50% | 0% |
| Frequency of quantification (%) | 0% | 0% | 0% | 0% | 0% | 0% | 28.7% | 0% |
| Range ppb* |  |  |  |  |  |  | 0.06-0.62 |  |
| mean +/- SD ppb* |  |  |  |  |  |  | 0.27+/-0.24 |  |
| **Azoxystrobin in nectar**  (LOD 0.02 ppb, LOQ 0.06 ppb) | *N=13* | *N=6* | *N=14* | *N=7* | *N=16* | *N=13* | *N=17* | *N=9* |
| Frequency of detection (%) | 0% | 0% | 0% | 0% | 0% | 0% | 0% | 0% |
| Frequency of quantification (%) | 0% | 0% | 0% | 0% | 0% | 0% | 0% | 0% |
| Range ppb* |  |  |  |  |  |  |  |  |
| mean +/- SD ppb* |  |  |  |  |  |  |  |  |
| **Azoxystrobin in pollen**  (LOD 0.014 ppb, LOQ 0.042 ppb) | *N=8* | *N=6* | *N=12* | *N=5* | *N=10* | *N=9* | *N=14* | *N=5* |
| Frequency of detection (%) | 87.50% | 83.30% | 58.30% | 60% | 50% | 55.50% | 42.90% | 100% |
| Frequency of quantification (%) | 50% | 50% | 25% | 20% | 20% | 0% | 35.70% | 20% |
| Range ppb* | 0.06-0.21 | 0.11-0.27 | 0.1-0.24 | 0.23 | 0.04-0.06 |  | 0.05-2.1 | 0.37 |
| mean +/- SD ppb* | 0.13+/-0.07 | 0.17+/-0.09 | 0.16+/-0.07 | 0.23+/-0 | 0.05+/-0.01 |  | 0.53+/-0.88 | 0.37+/-0 |
| **Fludioxonil in nectar**  (LOD 0.3 ppb, LOQ 0.9 ppb) | *N=13* | *N=6* | *N=14* | *N=7* | *N=16* | *N=13* | *N=17* | *N=9* |
| Frequency of detection (%) | 7.7% | 0% | 7% | 6% | 6% | 0% | 6% | 0% |
| Frequency of quantification (%) | 0% | 0% | 0% | 0% | 0% | 0% | 0% | 0% |
| Range ppb* |  |  |  |  |  |  |  |  |
| mean +/- SD ppb* |  |  |  |  |  |  |  |  |
| **Fludioxonil in pollen**  (LOD 0.14 ppb, LOQ 0.42 ppb) | *N=8* | *N=6* | *N=12* | *N=5* | *N=10* | *N=9* | *N=14* | *N=5* |
| Frequency of detection (%) | 0% | 0% | 0% | 0% | 0% | 0% | 0% | 0% |
| Frequency of quantification (%) | 0% | 0% | 0% | 0% | 0% | 0% | 0% | 0% |
| Range ppb* |  |  |  |  |  |  |  |  |
| mean +/- SD ppb* |  |  |  |  |  |  |  |  |
